# Diffuse Large B-Cell Lymphoma Presenting with Bilateral Renal Masses and Hematuria: A Case Report

**DOI:** 10.4274/tjh.2015.0238

**Published:** 2016-05-16

**Authors:** Şiyar Erdoğmuş, Serkan Aktürk, Zeynep Kendi Çelebi, Saba Kiremitçi, Gülşah Kaygusuz, Namık Kemal Altınbaş, Evren Üstüner, Kenan Keven

**Affiliations:** 1 Ankara University Faculty of Medicine, Department of Nephrology, Ankara, Turkey; 2 Ankara University Faculty of Medicine, Department of Pathology, Ankara, Turkey; 3 Ankara University Faculty of Medicine, Department of Radiology, Ankara, Turkey

**Keywords:** Acute kidney injury, Hematuria, Lymphoma, Renal biopsy, Renal masses

## Abstract

Renal involvement is most often seen in conjunction with multisystemic, disseminated lymphoma either by direct extension from a retroperitoneal mass or via hematogenous spread. Primary lymphoma of the kidney is not a common entity and it is a controversial issue on account of the absence of lymphatic tissues in the normal kidney. In this case report, we describe a 19-year-old male with hematuria, acute kidney injury, and bilateral renal masses due to massive lymphomatous infiltration of the kidneys, which was diagnosed as diffuse large B-cell non-Hodgkin lymphoma by Tru-Cut biopsy.

## Abstract (tr)

Lenfomada renal tutulum sıklıkla multisistemik olarak, retroperitoneal kitlenin direkt komşuluğu yoluyla veya hematojen yayılım şeklinde ortaya çıkar. Böbrekte lenfatik doku yokluğu nedeniyle primer böbrek lenfoması nadir görülen ve tartışmalı bir durumdur. Bu olguda; hematüri, akut böbrek hasarı ve böbreklerin masif lenfomatöz infiltrasyonuna bağlı bilateral renal kitle ile prezente olmuş ve böbrek biyopsisi ile diffüz büyük B hücreli non-Hodgkin lenfoma tanısı almış 19 yaşında erkek hasta sunulmaktadır.

## INTRODUCTION

Primary renal lymphoma (PRL) is a very rare disease and a controversial issue because the kidneys do not normally contain lymphatic tissue [1,2,3,4,5]. In general, renal lymphoma is most often seen along with dissemination of systemic disease and clinically silent. Occasionally, patients present nonspecific signs and symptoms as well flank pain, weight loss, fever, hematuria, and palpable mass [6]. Acute renal failure due to lymphomatous infiltration of the kidney has rarely been reported [7,8,9,10,11,12,13,14,15]. In this case report, we describe a 19-year-old male who presented with painless hematuria, acute kidney injury, and bilateral renal masses.

## CASE PRESENTATION

A 19-year-old male patient was admitted to the Nephrology Department of Ankara University Faculty of Medicine due to painless hematuria, bilateral renal masses, and acute kidney injury for further investigations. He was first evaluated at another center for hematuria. There was one episode of hematuria, which had subsided spontaneously. Abdominal ultrasonography had revealed bilateral diffuse renal masses and the patient was referred to our center for further examination.

On admission, there was no history of fever, weight loss, night ssweats and any other health problem. The patient’s physical examination findings were unremarkable. In particular, there was no peripheral lymphadenopathy or hepatosplenomegaly. Also the kidneys were not palpable. Laboratory tests revealed white blood cell count of 7.7x109/L, hemoglobin of 11.6 g/dL, platelet count of 315x109/L, serum blood urea nitrogen of 17 mg/dL, serum creatinine of 1.5 mg/dL (normal range: 0.5-0.9), serum uric acid of 7.6 mg/dL (normal range: 2.4-5.7), serum lactate dehydrogenase of 1042 U/L (normal range: 125-220), and serum ferritin of 749 ng/mL (normal range: 11-307). Erythrocyte sedimentation rate was 52 mm/h and C-reactive protein level was 27.5 mg/L (normal: <3). Urinalysis showed density of 1010, pH of 5.5, protein of 15 mg/dL, and glucose negative, and urine microscopy showed 4 leukocytes and 3 erythrocytes per high-power field. Viral serology tests were negative. C3 and C4 as well as quantitative immunoglobulin levels were all within normal limits with negative antinuclear antibody and anti-neutrophil cytoplasmic antibody tests. Urinary ultrasonography demonstrated bilaterally enlarged kidneys without obstruction (right: 16x8 cm, left: 15.5x8 cm) and numerous solid hypoechoic nodular cortical masses in both kidneys (largest of 6.5x5.5 cm in size, numerous variably sized masses) with perirenal and paraaortic multiple enlarged lymph nodes (largest <2.5 cm in size).

**Figure 1:**
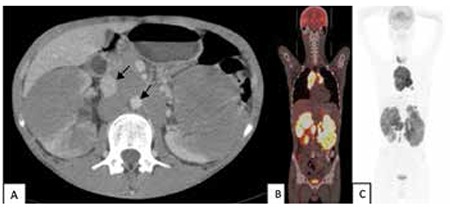
Computed tomography with intravenous contrast reveals enlargement of both kidneys with bilateral renal masses and paraaortic, paracaval lymph nodes (arrows) (A); 18F-fluorodeoxyglucose positron emission tomography-computed tomography fusion images showed very intense diffuse fluorodeoxyglucose uptake in bilaterally enlarged kidneys (B); maximum intensity projection images of positron emission tomography-computed tomography scan demonstrated multifocal increased 18F-fluorodeoxyglucose uptake in the thyroid, mediastinum, and kidneys (C).

Contrast-enhanced computerized tomography (CT) scanning of the abdomen confirmed bilaterally enlarged kidneys, bilateral variably sized multiple hypodense renal masses, and paraaortic, paracaval multiple enlarged lymph nodes (Figure 1A). In addition, the liver was slightly enlarged with normal parenchyma while the size of the spleen and parenchyma was normal.

Thereafter, ^18^F-fluorodeoxyglucose positron emission tomography-computed tomography (18F-FDG PET-CT) was performed for staging and its role in the differential diagnosis. It was performed to examine the entire body, revealing an abnormal accumulation in the thyroid gland, anterior mediastinum, bilateral hilar, right parasternal lymph node, left subdiaphragmatic lymph node, bilateral renal cortices, and multiple abdominal paraaortic, paracaval, and aortocaval lymph nodes (Figures 1B and 1C).

**Figure 2:**
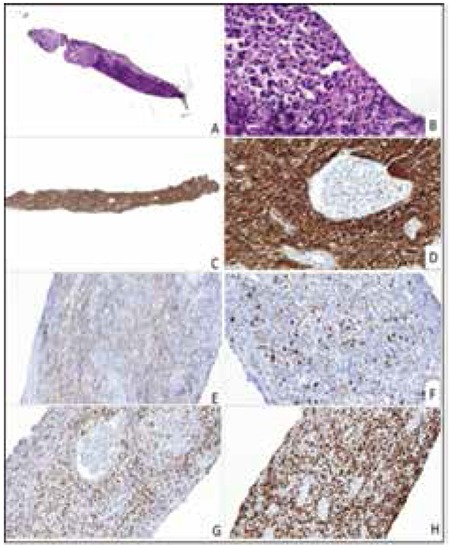
Atypical large lymphoid cells infiltrating the renal interstitium (A, B) (H&E, 65x, 830x), immunohistochemical CD20 (C, D) (37x, 479x), CD10 (E) (506x), and BCL6 expression of the neoplastic cells (G) (333x). MUM1 was negative (F) (397x). Ki67 immunostaining showed high proliferation index (H) (282x).

A percutaneous tru-cut biopsy of the kidney was performed and histopathological examination showed extensive infiltration of the renal parenchyma by atypical lymphoid cells (Figures 2A and 2B). Immunohistochemical studies demonstrated positive staining of the neoplastic cells for CD20, CD10, bcl-6 (Figures 2C, 2D, 2E, and 2G) and negative results for MUM1 (Figure 2F) and Bcl-2. The ki-67 proliferation index of neoplastic cells was 80% (Figure 2H). EBER in situ hybridization was negative. A diagnosis of diffuse large B-cell non-Hodgkin lymphoma (NHL) was made. To exclude lymphoma involvement of the bone marrow, the patient underwent bone marrow biopsy and there was not bone marrow infiltration.

Subsequently, the patient was transferred to the department of hematology for treatment and chemotherapy regimen as well; cyclophosphamide, adriamycin, vincristine, prednisolone, and rituximab (CHOP+R) were started. After a cycle of chemotherapy, the patient’s renal functions returned to normal.

## DISCUSSION AND REVIEW OF THE LITERATURE

Extranodal spread of lymphoma often affects the gastrointestinal tract, liver, central nervous system, genitourinary tract (e.g., kidney, testis, ovary), bone, bone marrow, lungs, breast, thyroid, and skin [16,17]. The most common site of genitourinary involvement is the kidney, usually in patients with intermediate and high-grade B-cell type NHL or American Burkitt lymphoma. Additionally, extranodal involvement of lymphoma is seen in most patients at the time of diagnosis [18,19,20]. Primary renal NHL is not a common clinical entity and it is a disputed issue owing to the absence of lymphoid tissue in normal kidneys. Malbrain et al. [8] suggested the use of some criteria for the diagnosis of PRL. These include: 1) Renal failure as the initial presentation, 2) Bilateral enlargement of the kidneys without obstruction and other organ or nodal involvement, 3) Diagnosis only made by renal biopsy, 4) Absence of other causes of renal failure, and 5) Rapid improvement of renal function after radiotherapy or systemic chemotherapy. Our patient presented with one episode of hematuria, which had subsided spontaneously, and bilateral involvement of the kidneys. His blood tests showed slight renal function impairment (serum creatinine: 1.5 mg/dL). Furthermore, there were not any causes of renal failure such as obstructive uropathy, hypercalcemia, uric acid nephropathy, volume depletion, and nephrotoxic drugs. The diagnosis of diffuse large B-cell NHL was established by Tru-Cut biopsy of the kidney. After a cycle of chemotherapy, his creatinine level returned to normal. The patient had massive infiltration of the kidneys along with thyroid gland infiltration, mediastinal involvement, and multiple enlarged lymph nodes in different sites. Consequently, our patient fulfilled four of the above criteria, and if we had used these criteria, we could not have accepted the diagnosis of PRL.

A variety of benign and malignant masses can involve the kidneys in a bilateral fashion. For example, metastatic disease, lymphoproliferative disorders, adult polycystic kidney disease, and angiomyolipoma are more commonly found in a bilateral fashion compared with transitional cell carcinomas or oncocytomas [21]. Several radiologic options exist for the evaluation of renal masses, although CT scan is the most common imaging modality used for the evaluation of renal lymphoma. Usually, definitive diagnosis of renal masses is made by renal biopsy. Urban and Fishman [22] reported that the most commonly encountered pattern of involvement in patients with renal lymphoma is multiple renal masses that are mostly bilateral. Other patterns include renal invasion from contiguous retroperitoneal tumors, perirenal masses, and diffuse renal infiltration [18,22,23]. Our patient presented with bilateral renal enlargement and renal masses in ultrasonography and CT scan. The patient’s diagnosis was made by ultrasonography-guided renal biopsy.

Whole-body imaging with 18F-FDG PET-CT is obligatory to assess the extent of disease by detecting unexpected extranodal sites of disease or for exclusion of disease in the presence of nonspecific extranodal CT findings [24,25]. In the present case, in addition to the CT findings, involvement of the thyroid gland and mediastinum was determined by 18F-FDG PET-CT.

In conclusion, in this case, we present bilateral renal masses due to massive lymphomatous infiltration of the kidneys, which was diagnosed as diffuse large B-cell NHL by tru-cut biopsy. The presence of extrarenal involvement in the thyroid gland and mediastinal, hilar, subcarinal, and multiple abdominal lymph nodes made the diagnosis of PRL debatable. Physicians should be aware of the probability of lymphoma in the differential diagnosis of renal masses.

## Ethics

Informed Consent: It was taken.
